# Dynamic Interactions Among Sleep Duration, Cognitive Function, and Depressive Symptoms in Middle-Aged and Older Chinese Adults: Temporal Network Analysis From CHARLS

**DOI:** 10.2196/76210

**Published:** 2025-09-16

**Authors:** Furong Chen, Jiaying Li, Junchen Guo, Ying Xiong, Zengjie Ye

**Affiliations:** 1 School of Nursing, Guangzhou Medical University, No.1 Xinzao Road, Panyu District, Guangzhou, Guangdong Province, 511436, China, 86 02037103000; 2 The Nethersole School of Nursing, Faculty of Medicine, The Chinese University of Hong Kong, Hong Kong SAR, China; 3 School of Nursing, University of Wollongong, Wollongong, Australia; 4 School of Nursing, Guangzhou University of Chinese Medicine, Guangzhou, Guangdong Province, China

**Keywords:** CHARLS, sleep, cognition, depression, temporal network analysis, middle-aged and older people, China Health and Retirement Longitudinal Study

## Abstract

**Background:**

While bidirectional associations among sleep duration, cognitive function, and depression are established, the symptom-level temporal interactions among these factors in China’s aging population, which is experiencing unprecedented growth, remain poorly characterized.

**Objective:**

We aim to use a novel temporal network analysis to clarify these dynamics and guide targeted interventions, with a focus on sex-specific dynamic pathways.

**Methods:**

We conducted a longitudinal temporal network analysis on 3136 Chinese adults aged ≥45 years from the China Health and Retirement Longitudinal Study (CHARLS) across 5 waves (2011, 2013, 2015, 2018, and 2020). A graphical vector autoregressive (GVAR) model delineated the interdependencies among sleep duration, cognitive performance (assessed via the Mini-Mental State Examination [MMSE]), and depressive symptoms (evaluated with the 10-item Center for Epidemiologic Studies Depression Scale [CESD-10]). We also examined sex-specific differences in network structures.

**Results:**

The symptom “bothered” was found to predict all other CESD-10 symptoms. There were significant predictive links between sleep and the CESD-10 node (ie, bothered, drained, and depressed), along with sleep and the MMSE functions (ie, numerical ability). Furthermore, sleep duration served as a bridge between depression symptoms and cognitive functions. There were significant differences in longitudinal network structure between sexes. Sex-specific analyses revealed distinct network patterns. Among female participants, the “bothered” node significantly predicted several outcomes over time. In contrast, the temporal network for male participants was sparser, with the “stuck” node in the depression domain being predominantly influenced by other nodes.

**Conclusions:**

Our study revealed that emotional distress, especially the “bothered” symptom, plays a central role in depressive symptoms and cognitive decline. The bridging effect of short sleep duration underscores the potential of interventions targeting both sleep and emotional distress for alleviating depressive symptoms and delaying cognitive deterioration in older adults.

## Introduction

The population is aging at an unprecedented rate in China, driven by socioeconomic advancements and improved health care [1]. This demographic shift poses critical challenges to sustaining the cognitive well-being and mental health of older adults [2]. Studies have reported that the prevalence of depressive symptoms in the middle-aged and older population in China ranges from 24.1% to 31.8% [3,4], and the prevalence of cognitive impairment is 26.7% [5]. This population also experiences more frequent and severe sleep issues [6]. For the middle-aged and older population, these 3 elements could significantly affect their physical and psychological status and quality of life. Therefore, clarification of the relationship among the 3 could facilitate the development of targeted interventions and improvement of health care policies.

Impaired cognition and sleep disturbances are prevalent in aging populations, and accumulating evidence suggests that sleep deficits may mediate up to 15% of cognitive decline [7-9]. In addition, depression is a common comorbidity in older adults, which further exacerbates cognitive risks [10]. The dual impact of sleep deprivation and mood dysregulation may compromise hippocampal-dependent cognition, while sleep fragmentation can further amplify the hyperactivity of the limbic system characteristic of depression [11-13]. Despite these advances, several limitations persist in existing studies. The relationship between depression and cognitive impairment is complex and bidirectional, yet its predictive nature remains unclear. Moreover, most studies have relied on cross-sectional designs, which do not capture the temporal directionality of associations among sleep, cognition, and depression. While studies by Zhou et al [11] and Sun et al [14] have investigated bidirectional cognition-depression links and sleep-depression interactions in Chinese cohorts, the potential simultaneous interactions and between-subject effects among these 3 variables remain insufficiently explored. Furthermore, although previous research has investigated sex differences in these relationships [15-18], further investigation into sex-based differences in middle-aged and older populations using longitudinal predictive frameworks is needed to address gaps in current research.

Traditional analytical approaches, while establishing bidirectional associations among sleep, cognition, and depression, often fail to capture symptom-level dynamics. Temporal network analysis, a novel approach in psychosomatic research, offers a dynamic framework to model bidirectional and triadic relationships over time [19]. Unlike traditional cross-lagged panel models, temporal network analysis distinguishes between within-person (lagged) and between-person (contemporaneous) effects, offering greater mechanistic clarity than conventional cross-sectional or longitudinal designs [20]. In this study, temporal network analysis enables us to examine how sleep, cognitive function, and depressive symptoms interact and predict each other over time, while also disentangling within-person and between-person effects.

This study aims to explore the dynamic connections between sleep duration, cognitive function, and depressive symptoms in middle-aged and older Chinese adults using large-scale longitudinal data and temporal network analysis. We hypothesized that (1) emotional distress is associated with increased depression symptoms, (2) sleep duration acts as a link between depression and cognitive decline, and (3) there are significant sex-specific differences in those temporal networks.

## Methods

### Study Design and Data Source

This temporal network analysis used longitudinal data from the China Health and Retirement Longitudinal Study (CHARLS), a nationally representative survey of Chinese adults aged 45 years and older. Funded by Peking University, CHARLS uses a stratified, probability-proportional-to-size sampling method to ensure comprehensive geographic coverage. The survey spanned 28 provinces, including autonomous regions and municipalities directly governed by the central government, covering 150 counties and 450 communities.

This analysis used data from 5 waves collected in 2011, 2013, 2015, 2018, and 2020, initially recruiting 17,705 participants in 2011. Participants were excluded if they had (1) missing baseline data on cognition or depression, (2) missing demographic variables, (3) age <45 years at baseline, and (4) missing follow-up data on cognition and depression.

### Measurement

#### Demographic Variables

The participants provided information regarding their age, sex, education level, retirement status, and marital status.

#### Depression Assessment

Depressive symptoms were measured using the 10-item Center for Epidemiologic Studies Depression Scale (CESD-10) [21]. This instrument was well validated, demonstrating excellent interrater reliability and specificity in Chinese middle-aged and older Chinese adults [22,23]. The scale comprised 10 items, with the final score calculated as the sum of individual item scores, ranging from 0 to 30. A total score of 10 or greater was used to indicate the presence of depressive symptoms. Measurements were conducted by trained CHARLS interviewers during face-to-face home visits as part of the core questionnaire.

#### Cognition Assessment

Cognitive function was evaluated using the Mini-Mental State Examination (MMSE) [24]. This assessment focused on 2 primary domains: episodic memory and mental status. Episodic memory was assessed using a 10-word recall test. Participants were read a random word list and asked to immediately recall as many words as possible (immediate recall score: 0‐10). After a 10-minute interval, delayed recall of the same list was tested (delayed recall score: 0‐10). The episodic memory score (range 0‐10) was calculated as the average of these 2 trials. Mental intactness was evaluated using adapted components from the Telephone Interview for Cognitive Status (TICS). This included serial-7 subtractions from 100 (maximum 5 trials), temporal orientation (date, day, and season), and intersecting pentagon copying. Responses were summed into a composite score ranging 0‐11. The global cognitive score (range 0‐21) represented the sum of episodic memory and mental intactness scores. All cognitive tests were administered directly by trained CHARLS interviewers during in-home visits following standardized protocols.

#### Sleep Duration

Nighttime sleep duration was assessed by asking respondents, “On average, how many hours per night did you actually sleep in the past month?” Responses were recorded verbatim by interviewers during face-to-face home visits.

### Statistical Analyses

Overall, the analytical approach comprised several key steps designed to rigorously model and interpret the longitudinal dynamics among sleep duration, cognitive function, and depressive symptoms. We first preprocessed the data to ensure normality and stationarity, then selected appropriate nodes to represent distinct constructs, and finally used a graphical vector autoregressive (GVAR) model for network estimation. All analyses were conducted using R (version 4.4.1; R Core Team).

### Data Preparation

Before performing the network analyses, we undertook a series of data preprocessing steps to ensure that the assumptions underlying dynamic network models were satisfied [25,26]. First, we confirmed that each participant provided enough observations for the longitudinal analysis. Next, to verify that our data were suitable for parametric analysis, we applied the Shapiro-Wilk normality test. This test indicated that the distribution of our key variables did not deviate substantially from normality; given the large sample size, this justified the use of methods that assume normally distributed data. Finally, we evaluated the stationarity of the time series data using the Kwiatkowski-Phillips-Schmidt-Shin (KPSS) test [26]. Stationarity was essential for dynamic models because it ensures that the statistical properties of the variables (such as mean and variance) remain constant over time. In our analysis, the KPSS test results were all nonsignificant (False), confirming that the data met the stationarity assumption required for reliable longitudinal analysis.

### Node Selection via Redundancy Checking

First, to avoid redundancy, we used the 5 subscales of the MMSE as separate nodes in the network analysis, rather than a single item, since the entries under these dimensions share similar characteristics [27]. Second, each depression symptom and the sleep duration score were treated as individual nodes to provide a comprehensive representation of these factors. In total, 16 nodes (10 depression symptoms, 5 MMSE subscales, and 1 sleep scale) were included in the network based on the available data. Node redundancy was assessed at each time point using the goldbricker function [27], with no suggested reductions. The node abbreviations, description, and items for CESD-10, MMSE, and Sleep are provided in Table S1 in Multimedia Appendix 1.

### Network Estimation and Visualization

The GVAR model was constructed using the psychonetrics package to explore the longitudinal relationships between the study variables [28].

The panel GVAR captures temporal dependencies as partial correlations between the deviations from the individual mean of a variable at one time point and the deviations at the following time point, while adjusting for all variables at the preceding time point. This creates a matrix of regression coefficients that can be used to plot a directed lag-1 network (ie, the temporal or longitudinal network), which represents the generalized temporal within-subject effects between variables [28]. The panel GVAR also estimates 2 additional networks through 2 Gaussian graphical models (GGMs)—a contemporaneous network that interprets the relationships among variables within a person at a specific time point, adjusting for temporal effects, and a between-person network that captures associations between the stationary means across all participants.

The GVAR model was estimated using full information maximum likelihood (FIML) estimation. We searched for the optimal model (ie, pruned model) by minimizing the Bayesian information criterion and threshold at *α*=.05. To assess model fit, we used the confirmatory fit index (CFI) and the Tucker-Lewis index (TLI) and the root mean squared error of approximation (RMSEA), with values >0.85 (CFI and TLI) and <0.05 (RMSEA) indicating good fit [29].

The psychonetrics package uses the qgraph package [30] to create graphical network models displaying the estimated coefficients. For both contemporaneous and between-subject networks, the conservative “AND-rule” was used to retain significant edges, meaning that an edge was included only if both regressions it relied on were significant. Node centrality is interpreted through visual inspection of the network structures [31].

### Ethical Considerations

This study used publicly available data from the CHARLS survey, without any new sample recruitment or additional data collection. In that survey, informed consent was obtained from all participants. During the face-to-face household interviews, trained interviewers thoroughly explained the study objectives, interview content, potential risks and benefits, data confidentiality protocols, and the voluntary nature of participation (including the right to refuse or withdraw at any stage). Participants provided written informed consent. For participants who were illiterate, the consent form was read aloud in the presence of a witness, and participants provided a fingerprint or signature as confirmation. The CHARLS study underwent a thorough review and received approval from the Ethics Review Board of Peking University (IRB00001052-11015), ensuring adherence to the highest ethical standards.

The analysis in this study used this de-identified, publicly available data that had already received institutional ethics approval for the original data collection. Our analysis falls within the scope of research purposes for which the data were originally collected and approved. In addition, co-author Junchen Guo obtained formal authorization to access and use the CHARLS dataset through the official CHARLS website. All data usage complies with the CHARLS dataset’s terms of use and citation requirements established by the National School of Development, Peking University.

## Results

### Demographic Characteristics of Participants

**Figure 1.**
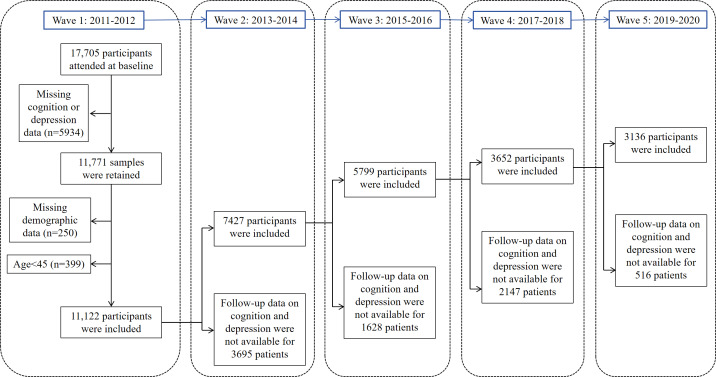
Study flowchart. The figure shows 2 different types of missing data: Within-wave missing data: for example, in Wave 3, 1628 participants had missing cognition or depression data within that wave. Between-wave attrition: 2147 participants were lost between Wave 3 and Wave 4. The mathematics is correct: Wave 3 (5799) - attrition to Wave 4 (2147) = Wave 4 participants (3652). Similarly, Wave 4 (3652) - attrition to Wave 5 (516) = Wave 5 participants (3136). The numbers we referenced (1628 for Wave 3) represent missing data within that specific wave, not the attrition between waves.

After applying the inclusion criteria, the final analytic sample comprised 3136 individuals (refer to Figure 1 for the screening flowchart). Among the 3136 eligible individuals, ages ranged from 45 to 81 years, with a mean age of 55.73 (SD 7.47) years. In addition, 56.8% (1782/3136) of the participants were male. Detailed demographics and characteristics are presented in Table 1.

**Table 1.** Demographics and characteristics of the sample (N=3136).

Characteristics	Values
Age (years), mean (SD)	55.73 (7.47)
Sex, n (%)	
Male	1782 (56.8)
Female	1354 (43.2)
Marital status, n (%)	
Married	2963 (94.5)
Unmarried	12 (0.4)
Divorced	22 (0.7)
Widowed	139 (4.4)
Education, n (%)	
Primary school and below	1363 (43.5)
Junior high school	1098 (35)
High school or Middle professional school	598 (19.1)
University and above	77 (2.4)
Retirement status	
Yes	838 (26.7)
No	2298 (73.3)

### Model Fitting Results

We selected the step-up model as our final framework, with detailed fit indices presented in Table 2. The model demonstrated acceptable fit to the data (*χ*^2^_3135_=9373.40; *P*<.001; RMSEA=0.025; 95% CI 0.025-0.026; CFI=0.89; TLI=0.89). Complete parameter matrices for the estimated temporal, contemporaneous, and between-subject networks are provided in Tables S2-S4 in Multimedia Appendix 1, and Figures S1-S3 in Multimedia Appendices 2-4 illustrate the node centrality of these networks.

**Table 2.** Model fit indices across model types.

Model type	*χ*² (*df*)	RMSEA^a^ (95% CI)	CFI^b^	TLI^c^	AIC^d^	BIC^e^
Baseline	7712.06 (2776)	0.024 (0.023‐0.024)	0.89	0.87	656,172.84	659,464.43
Pruned (*α*=.05)	11,638.07 (3171)	0.029 (0.029‐0.030)	0.81	0.80	659,308.86	660,210.41
Stepup	9373.40 (3135)	0.025 (0.025‐0.026)	0.89	0.89	657,116.18	658,235.56

a RMSEA: root mean squared error of approximation.

b CFI: confirmatory fit index.

c TLI: Tucker-Lewis index.

d AIC: Akaike information criterion.

e BIC: Bayesian information criterion.

### Temporal Network

**Figure 2.**
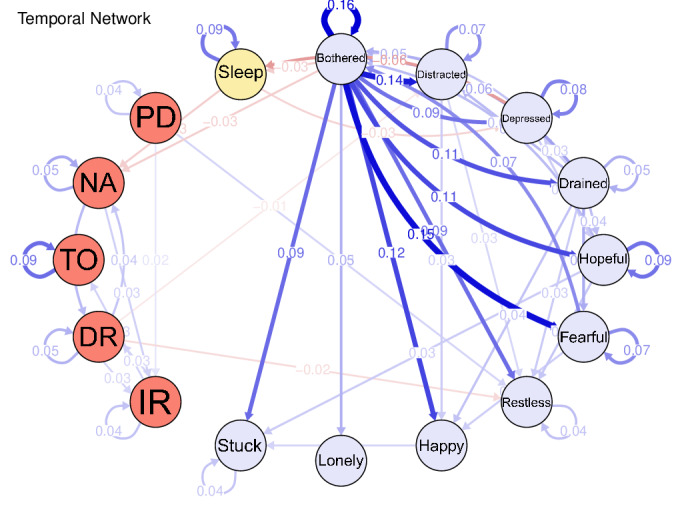
The temporal network.

Figure 2 illustrates the temporal network, highlighting the within-subject temporal predictions between nodes. Bothered (“I was bothered by things that usually don’t bother me.”) was the core node, predicting all other nodes in the CESD-10 scale positively and significantly. Moreover, bothered had a negative and significant effect on “NA” (numerical ability, *β*=−.030) in the MMSE scale. Higher Bothered (*β*=−.031) and Depressed (“I felt depressed.”, *β*=−.062) predicted shorter sleep duration. In addition, longer sleep duration predicted lower levels of NA (*β*=−.031).

### Contemporaneous Network

**Figure 3.**
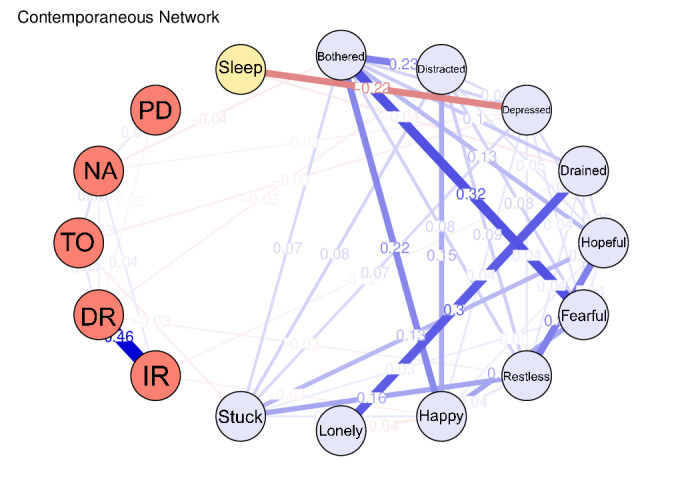
The contemporaneous network.

The contemporaneous network (Figure 3; Table S3 in Multimedia Appendix 1) reflects cross-sectional associations at individual time points after adjusting for temporal effects. Most connections occurred within the same measurement domain; however, 2 notable cross-domain links were observed. Specifically, the “depressed” node in the depression symptom group was negatively correlated with the time orientation node (*r*=−0.029), the NA node in cognition (*r*=−0.023), and the sleep duration node (*r*=−0.221). In addition, drained (“I felt that everything I did was an effort.”) in CESD-10 correlated negatively with immediate recall (*r*=−0.023) in MMSE and sleep duration (*r*=−0.038).

### Between-Subject Network

**Figure 4.**
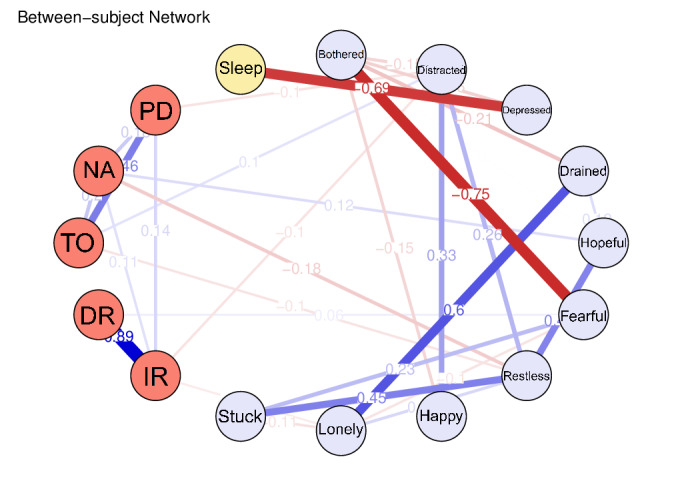
The between-subject network.

In the between-subject network (Figure 4), the “bothered” node exhibited the most extensive interscale associations. For example, this node was negatively correlated with the “fearful” (*r*=−0.749) and “drained” (*r*=−0.208) nodes within the depression symptoms. In addition, the cognition “NA” (numerical ability) node was positively associated with the depression “hopeful” node (*r*=0.121) and negatively associated with the “restless” node (*r*=−0.179). Notably, the “sleep duration” node maintained a negative association with the depression “depressed” node (*r*=−0.692).

### Sex Differences

Sex-specific analyses revealed distinct network patterns. Among female participants (Multimedia Appendix 5), the “bothered” node significantly predicted several outcomes over time, including the “fearful” node (*β*=.149) in the depression domain. In contrast, the temporal network for male participants (Multimedia Appendix 6) was sparser, with the “stuck” node in the depression domain being predominantly influenced by other nodes. The contemporaneous networks (MultimediaAppendices 7  8) demonstrated similar cross-sectional linkages across sexes. Interestingly, the between-person network for males (Multimedia Appendix 9 for male participants; Multimedia Appendix 10 for female participants) exhibited atypical associations, whereby higher “bothered” scores were linked with greater levels of “happy” (*r*=0.374) and “hopeful” (*r*=0.270). Details are provided in Tables S5-S10 in Multimedia Appendix 1.

## Discussion

### Principal Findings

To the best of our knowledge, this is the first predictive longitudinal study of sleep, cognition, and depression in a large Chinese middle-aged and older population.

In our temporal network analysis, the node “bothered” emerged as central, significantly predicting all other depressive symptoms and showing a negative association with numerical ability—a key cognitive measure. This finding reinforces the central role of emotional distress in depression [32]. Although the corresponding *β* value is low, this reflects the nature of symptoms as collections of moment-by-moment experiences, with these micro-level processes forming the foundation of psychopathology. While the effect of a single instance may be limited, the cumulative reduction in “bothered” moments, whether through mindfulness or environmental adjustments, may lead to clinically meaningful improvements in negative affect.

Moreover, both “bothered” and “depressed” were negatively associated with shorter sleep duration, aligning with previous findings [33]. Intriguingly, longer sleep duration predicted lower numerical ability, perhaps because middle-aged and older individuals, despite extended sleep periods, often experience frequent nocturnal awakenings that compromise sleep quality [34]. This impaired sleep quality may contribute to accelerated atrophy in the frontal, temporal, and parietal regions—areas critical for memory and numerical processing [35,36], ultimately leading to cognitive decline [37].

In addition, these results suggest that sleep may serve as a crucial intermediary between depression and cognitive function. Depression can adversely affect cognition directly or indirectly through elevated harmful cytokine levels [38,39] and reduced hippocampal volume [40]. Previous studies have highlighted that the association between sleep duration and cognitive function is substantially influenced by the severity of depressive symptoms [12,13,41]. Given that depressive symptoms are closely associated with sleep disturbances [42] and that inadequate sleep may lower the secretion of brain-derived neurotrophic factor, thereby impairing cognitive ability [43]. Similar findings have been observed in US adolescent populations, showing that functional connectivity between the cortex and basal ganglia mediates the effects of insufficient sleep on depression and thought problems, while structural properties of the anterior temporal lobe mediate its effects on crystallized intelligence [44]. Therefore, improving sleep quality might not only alleviate emotional distress and depressive symptoms but also play an important role in delaying cognitive decline.

The contemporaneous network analysis revealed that depressive symptoms not only predict other symptoms over time but also exert an immediate influence on cognitive function and sleep quality. These findings reinforce the notion that depression is closely linked with declines in memory, attention, and overall cognitive performance [45]. A cohort study of older adults in the United Kingdom similarly demonstrated that reduced sleep was associated with depression and that baseline depression predicted subsequent sleep reduction [46]. Moreover, depression appears to diminish an individual’s adaptability to external changes, thereby exacerbating sleep disturbances [42]. In the between-subject network, the “bothered” node emerged as a critical hub connecting symptom dissemination across individuals, suggesting that social factors may influence the spread of emotional distress among middle-aged and older adults [47].

In the temporal network specific to females, the “bothered” node was the most central emotion, significantly impacting other depressive symptoms. This observation aligns with evidence that women tend to express emotions more readily and may be more vulnerable to negative affective states [48]. These patterns are further amplified by Chinese cultural norms of emotional labor, where women bear primary responsibility for familial emotional regulation, intensifying affective sensitivity through sustained practice. Conversely, in men, depressive symptoms appeared to manifest more independently, potentially reflecting a tendency toward emotional suppression or less overt emotional expression [49]. This dissociation may stem from Confucian masculinity norms that stigmatize emotional disclosure while sanctioning somatic codification of distress [50]. The difference can be explained by brain structure differences [51]. Variations in hippocampal volume and corpus callosum structure have been reported between sexes [52]. These differences might affect cognitive processing and interhemispheric communication, further modulating how emotional information is integrated and expressed [53].

In women, enhanced reactivity within the limbic system, particularly in the amygdala, and stronger connectivity between the amygdala and prefrontal cortex have been associated with heightened sensitivity to emotional stimuli and increased vulnerability to mood disturbances [54]. By contrast, men often exhibit differences in the structure and function of the prefrontal cortex and anterior cingulate cortex, which may confer a greater capacity for emotional regulation and influence the more discrete manifestation of depressive symptoms [55]. Notably, the between-subject network for males revealed instances where stress or emotional distress was associated with positive emotional responses. This phenomenon may be attributable to sex-specific emotion regulation strategies, such as self-encouragement or attentional shifting [56], highlighting the potential benefit of incorporating positive emotion regulation techniques in interventions targeted at Chinese men experiencing emotional distress.

The temporal dynamics observed in this network analysis carry significant implications for public health strategies targeting the Chinese aging population. Given the centrality of sleep duration in driving depressive symptoms and cognitive decline, community health centers could prioritize scalable sleep screening protocols, such as incorporating the Pittsburgh Sleep Quality Index (PSQI) into annual geriatric check-ups to identify high-risk individuals before downstream mental health deterioration occurs. Building on the bidirectional sleep-depression pathway, we further advocate for training primary care physicians in brief behavioral interventions for insomnia, leveraging the Chinese tiered health care system to deliver stepped-care models where digital platforms (eg, WeChat-based sleep trackers) provide first-line support, with psychiatric referrals reserved for complex cases. Crucially, the mediating role of cognitive function suggests that integrating cognitive preservation components into existing sleep programs, particularly for those with mild cognitive impairment, may amplify intervention benefits.

This study has several strengths. First, we examined both intraindividual and interindividual longitudinal networks for sleep duration, cognitive function, and depression symptoms. This approach addresses a growing need for research at the symptom level, representing a shift from traditional cross-sectional studies to analyses of complex, dynamic systems within individuals and across subpopulations. Second, we extended previous research to examine sex-specific network patterns in a Chinese context. Third, our innovative integration of sleep duration, cognition, and depression into a unified network analysis has enriched our understanding of the dynamic relationships among these factors, which is critical for mental well‐being and cognitive health in the aging population.

### Limitations

However, our study has limitations that offer directions for future research. First, the current data do not capture immediate variable relationships; future studies might use ecological momentary assessment to more accurately capture dynamic changes in sleep, cognition, and depression within natural environments [57]. Second, another limitation is the use of a single-item sleep duration measure, which does not capture sleep architecture or the presence of sleep disorders. Future network studies should prioritize multimodal sleep assessments (eg, actigraphy combined with clinical screening tools) to enhance measurement accuracy. Third, the sources of sex-specific network heterogeneity remain unclear. It is uncertain whether these differences stem solely from inherent group characteristics or from a combination of group differences and other psychosocial factors. Prospective experimental designs over extended periods are needed to clarify the causality of these sex differences. In addition, statistical comparisons of sex-stratified networks were not performed due to the absence of validated methods for testing longitudinal network invariance [58]. Finally, replicating and extending this study in a Western context would help determine whether the interaction patterns among sleep, cognition, and depression observed in Chinese middle-aged and older adults are consistent across cultural settings.

### Conclusions

This study pioneers the investigation of predictive relationships among sleep duration, cognitive function, and depression in a large cohort of middle-aged and older Chinese individuals. Our findings indicate that emotional distress—most notably the “bothered” symptom—plays a central role in predicting depressive symptoms and impairing cognitive function, while poor sleep duration serves as a critical link between depression and cognitive decline. These results emphasize the importance of addressing emotional distress and improving sleep duration as potential strategies to mitigate depressive symptoms and slow cognitive decline in older adults.

## Supplementary material

10.2196/76210 Multimedia Appendix 1 Node abbreviation index, complete network analysis results, with sex-specific results for female and male groups.

10.2196/76210 Multimedia Appendix 2 Expected Influence of network nodes among temporal network.

10.2196/76210 Multimedia Appendix 3 Centrality of network nodes among contemporaneous network, including Expected Influence (A), Closeness (B), Strength (C), and Betweenness (D).

10.2196/76210 Multimedia Appendix 4 Centrality of network nodes among between-subject network, including Expected Influence (A), Closeness (B), Strength (C), and Betweenness (D).

10.2196/76210 Multimedia Appendix 5 Temporal network models for female participants.

10.2196/76210 Multimedia Appendix 6 Temporal network models for male participants.

10.2196/76210 Multimedia Appendix 7 Contemporaneous network models for male participants.

10.2196/76210 Multimedia Appendix 8 Contemporaneous network models for female participants.

10.2196/76210 Multimedia Appendix 9 Between-subject network models for male participants.

10.2196/76210 Multimedia Appendix 10 Between-subject network models for female participants.

## Abbreviations

CESD-10: 10-item Center for Epidemiologic Studies Depression Scale
CFI: confirmatory fit index
CHARLS: China Health and Retirement Longitudinal Study
FIML: full information maximum likelihood
GGMs: Gaussian graphical models
GVAR: graphical vector autoregressive
KPSS: Kwiatkowski-Phillips-Schmidt-Shin
MMSE: Mini-Mental State Examination
PSQI: Pittsburgh Sleep Quality Index
RMSEA: root mean squared error of approximation
TICS: Telephone Interview for Cognitive Status
TLI: Tucker-Lewis index
