# Supplementary material for: Dynamic Interactions Among Sleep Duration, Cognitive Function, and Depressive Symptoms in Middle-Aged and Older Chinese Adults: Temporal Network Analysis From CHARLS
Source: JMIR Aging. 2025 Sep 16;8:e76210. doi: 10.2196/76210 (PMC12440260; doi:10.2196/76210)
Supplement: Multimedia Appendix 1 [file aging-v8-e76210-s001.docx]

**Table S1.** Node abbreviation index.

| **Node** | **Description** | **Item** | **Scale** |
| --- | --- | --- | --- |
| Bothered | Unusually being bothered | 1. I was bothered by things that usually don't bother me. | CESD-10 |
| Distracted | Difficulty focusing | 1. I had trouble keeping my mind on what I was doing. |  |
| Depressed | Feeling depressed | 1. I felt depressed. |  |
| Drained | Struggling to cope | 1. I felt that everything I did was an effort. |  |
| Hopeful | Feeling hopeful | 1. I felt hopeful about the future. |  |
| Fearful | Feeling fearful | 1. I felt fearful. |  |
| Restless | Poor sleep quality | 1. My sleep was restless. |  |
| Happy | Feeling happy | 1. I was happy. |  |
| Lonely | Feeling lonely | 1. I felt lonely. |  |
| Stuck | Feeling stuck | 1. I could not "get going". |  |
| IR | Episodic memory | 1. Immediate recall (After investigators repeated a list of 10 random words, participants were demanded to recall as many words as possible immediately). | MMSE |
| DR |  | 1. Delayed recall (repeat the same words five minutes later). |  |
| TO | Mental intactness | 1. Time orientation {Including checking the date (year, month, day), the season of year and the day of week} |  |
| NA |  | 1. Numerical ability {Including serial subtraction of 7 from 100 (up to five times)}. |  |
| PD |  | 1. Picture drawing (redrawing a picture shown to the participants). |  |
| Sleep | Sleep duration | In the past month, on average, about how many hours per night did you actually fall asleep? | Questionnaire |

**Table S2.** Numeric results of Temporal network.

|  | 1 | 2 | 3 | 4 | 5 | 6 | 7 | 8 | 9 | 10 | 11 | 12 | 13 | 14 | 15 | 16 |
| --- | --- | --- | --- | --- | --- | --- | --- | --- | --- | --- | --- | --- | --- | --- | --- | --- |
| Bothered | 0.158 | 0.142 | 0.088 | 0.108 | 0.115 | 0.150 | 0.091 | 0.116 | 0.049 | 0.092 |  |  |  | -0.030 |  | -0.031 |
| Distracted | 0.050 | 0.069 |  |  |  |  | 0.025 | 0.032 |  |  |  |  |  |  |  |  |
| Depressed | 0.037 |  | 0.080 |  | 0.032 |  |  |  |  |  |  |  |  |  |  | -0.062 |
| Drained | 0.059 | 0.044 | 0.032 | 0.051 | 0.040 | 0.052 | 0.033 | 0.038 |  |  |  |  |  |  |  |  |
| Hopeful | 0.043 |  |  |  | 0.088 |  | 0.035 |  |  | 0.033 |  |  |  |  |  |  |
| Fearful | 0.073 |  |  |  |  | 0.072 |  | 0.031 |  |  |  |  |  |  |  |  |
| Restless |  |  |  |  |  |  | 0.040 |  |  |  |  |  |  |  |  |  |
| Happy |  |  |  |  |  |  |  |  |  | 0.027 |  |  |  |  |  |  |
| Lonely |  |  |  |  |  |  |  |  |  |  |  |  |  |  |  |  |
| Stuck |  |  |  |  |  |  |  |  |  | 0.036 |  |  |  |  |  |  |
| IR |  |  |  |  |  |  |  |  |  |  | 0.038 | 0.028 | 0.028 |  |  |  |
| DR |  | -0.015 |  |  |  |  | -0.023 |  |  |  | 0.026 | 0.048 |  | 0.036 |  |  |
| TO |  |  |  |  |  |  |  |  |  |  |  |  | 0.091 |  |  |  |
| NA |  |  |  |  |  |  |  |  |  |  | 0.029 | 0.043 |  | 0.055 |  |  |
| PD |  |  |  |  |  |  | 0.027 |  |  |  | 0.017 |  |  |  | 0.038 |  |
| Sleep |  |  | -0.027 |  |  |  |  |  |  |  |  |  |  | -0.031 |  | 0.085 |

The matrix shows how much each variable in the left column predicts the next lag-1 value of each variable in the top row. Diagonal numbers indicate edge weights of autoregressions.

**Table S3.** Numeric results of Contemporaneous network.

|  | 1 | 2 | 3 | 4 | 5 | 6 | 7 | 8 | 9 | 10 | 11 | 12 | 13 | 14 | 15 | 16 |
| --- | --- | --- | --- | --- | --- | --- | --- | --- | --- | --- | --- | --- | --- | --- | --- | --- |
| Bothered | 0 |  |  |  |  |  |  |  |  |  |  |  |  |  |  |  |
| Distracted | 0.231 | 0 |  |  |  |  |  |  |  |  |  |  |  |  |  |  |
| Depressed | 0.080 | 0.063 | 0 |  |  |  |  |  |  |  |  |  |  |  |  |  |
| Drained | 0.097 |  |  | 0 |  |  |  |  |  |  |  |  |  |  |  |  |
| Hopeful | 0.128 | 0.046 | 0.059 | 0.059 | 0 |  |  |  |  |  |  |  |  |  |  |  |
| Fearful | 0.317 | 0.080 | 0.045 | 0.053 | 0.037 | 0 |  |  |  |  |  |  |  |  |  |  |
| Restless | 0.083 | 0.088 | 0.041 | 0.041 | 0.231 |  | 0 |  |  |  |  |  |  |  |  |  |
| Happy | 0.217 | 0.152 | 0.039 |  | 0.029 | 0.161 | 0.044 | 0 |  |  |  |  |  |  |  |  |
| Lonely |  |  |  | 0.296 |  |  |  | -0.040 | 0 |  |  |  |  |  |  |  |
| Stuck | 0.069 | 0.080 | 0.069 |  | 0.132 | 0.033 | 0.162 | 0.040 |  | 0 |  |  |  |  |  |  |
| IR |  |  |  | -0.023 |  |  |  | -0.025 |  |  | 0 |  |  |  |  |  |
| DR |  | -0.027 |  |  |  |  | -0.027 |  |  |  | 0.464 | 0 |  |  |  |  |
| TO |  |  | -0.029 |  |  |  |  |  |  | -0.032 |  | 0.044 | 0 |  |  |  |
| NA | -0.036 |  | -0.023 |  |  |  |  |  |  |  | 0.037 | 0.030 | 0.057 | 0 |  |  |
| PD |  |  |  |  |  |  |  |  |  |  |  | 0.019 |  |  | 0 |  |
| Sleep |  |  | -0.221 | -0.038 |  |  |  |  |  |  |  |  |  |  |  | 0 |

Lower triangle indicates within-person partial correlations between variables in the leftmost column and variables in the topmost row within the same measurement occasion, after controlling for other variables and all temporal relationships.

**Table S4.** Numeric results of Between-subject network.

|  | 1 | 2 | 3 | 4 | 5 | 6 | 7 | 8 | 9 | 10 | 11 | 12 | 13 | 14 | 15 | 16 |
| --- | --- | --- | --- | --- | --- | --- | --- | --- | --- | --- | --- | --- | --- | --- | --- | --- |
| Bothered | 0 |  |  |  |  |  |  |  |  |  |  |  |  |  |  |  |
| Distracted | -0.150 | 0 |  |  |  |  |  |  |  |  |  |  |  |  |  |  |
| Depressed | -0.161 |  | 0 |  |  |  |  |  |  |  |  |  |  |  |  |  |
| Drained | -0.208 |  |  | 0 |  |  |  |  |  |  |  |  |  |  |  |  |
| Hopeful | -0.003 |  |  | 0.118 | 0 |  |  |  |  |  |  |  |  |  |  |  |
| Fearful | -0.749 |  |  |  |  | 0 |  |  |  |  |  |  |  |  |  |  |
| Restless |  | 0.261 |  |  | 0.435 |  | 0 |  |  |  |  |  |  |  |  |  |
| Happy | -0.147 | 0.334 |  |  |  |  |  | 0 |  |  |  |  |  |  |  |  |
| Lonely |  |  |  | 0.601 |  | -0.100 | 0.141 |  | 0 |  |  |  |  |  |  |  |
| Stuck |  |  |  |  |  | 0.235 | 0.447 |  | -0.109 | 0 |  |  |  |  |  |  |
| IR |  | -0.101 |  |  |  |  |  |  | -0.071 |  | 0 |  |  |  |  |  |
| DR |  |  |  |  |  | 0.060 |  |  |  |  | 0.895 | 0 |  |  |  |  |
| TO |  | 0.102 |  |  |  |  | -0.097 |  |  |  |  |  | 0 |  |  |  |
| NA |  |  |  |  | 0.121 |  | -0.179 |  |  |  | 0.110 |  | 0.195 | 0 |  |  |
| PD |  | -0.100 |  |  |  |  |  |  |  |  | 0.143 |  | 0.456 | 0.185 | 0 |  |
| Sleep |  |  | -0.692 |  |  |  |  |  |  |  |  |  |  |  |  | 0 |

Lower triangle indicates between-person partial correlations between variables in the leftmost column and variables in the topmost row within the same measurement occasion, after controlling for other variables.

**Table S5.** Numeric results of Temporal network for females.

|  | 1 | 2 | 3 | 4 | 5 | 6 | 7 | 8 | 9 | 10 | 11 | 12 | 13 | 14 | 15 | 16 |
| --- | --- | --- | --- | --- | --- | --- | --- | --- | --- | --- | --- | --- | --- | --- | --- | --- |
| Bothered | 0.158 | 0.142 | 0.088 | 0.108 | 0.115 | 0.150 | 0.091 | 0.116 | 0.049 | 0.092 |  |  |  | -0.030 |  | -0.031 |
| Distracted | 0.050 | 0.069 |  |  |  |  | 0.025 | 0.032 |  |  |  |  |  |  |  |  |
| Depressed | 0.037 |  | 0.080 |  | 0.032 |  |  |  |  |  |  |  |  |  |  | -0.062 |
| Drained | 0.059 | 0.044 | 0.032 | 0.051 | 0.040 | 0.052 | 0.033 | 0.038 |  |  |  |  |  |  |  |  |
| Hopeful | 0.043 |  |  |  | 0.088 |  | 0.035 |  |  | 0.033 |  |  |  |  |  |  |
| Fearful | 0.073 |  |  |  |  | 0.072 |  | 0.031 |  |  |  |  |  |  |  |  |
| Restless |  |  |  |  |  |  | 0.040 |  |  |  |  |  |  |  |  |  |
| Happy |  |  |  |  |  |  |  |  |  | 0.027 |  |  |  |  |  |  |
| Lonely |  |  |  |  |  |  |  |  |  |  |  |  |  |  |  |  |
| Stuck |  |  |  |  |  |  |  |  |  | 0.036 |  |  |  |  |  |  |
| IR |  |  |  |  |  |  |  |  |  |  | 0.038 | 0.028 | 0.028 |  |  |  |
| DR |  | -0.015 |  |  |  |  | -0.023 |  |  |  | 0.026 | 0.048 |  | 0.036 |  |  |
| TO |  |  |  |  |  |  |  |  |  |  |  |  | 0.091 |  |  |  |
| NA |  |  |  |  |  |  |  |  |  |  | 0.029 | 0.043 |  | 0.055 |  |  |
| PD |  |  |  |  |  |  | 0.027 |  |  |  | 0.017 |  |  |  | 0.038 |  |
| Sleep |  |  | -0.027 |  |  |  |  |  |  |  |  |  |  | -0.031 |  | 0.085 |

The matrix shows how much each variable in the left column predicts the next lag-1 value of each variable in the top row. Diagonal numbers indicate edge weights of autoregressions.

**Table S6.** Numeric results of Contemporaneous network for females.

|  | 1 | 2 | 3 | 4 | 5 | 6 | 7 | 8 | 9 | 10 | 11 | 12 | 13 | 14 | 15 | 16 |
| --- | --- | --- | --- | --- | --- | --- | --- | --- | --- | --- | --- | --- | --- | --- | --- | --- |
| Bothered | 0 |  |  |  |  |  |  |  |  |  |  |  |  |  |  |  |
| Distracted | 0.231 | 0 |  |  |  |  |  |  |  |  |  |  |  |  |  |  |
| Depressed | 0.080 | 0.063 | 0 |  |  |  |  |  |  |  |  |  |  |  |  |  |
| Drained | 0.097 |  |  | 0 |  |  |  |  |  |  |  |  |  |  |  |  |
| Hopeful | 0.128 | 0.046 | 0.059 | 0.059 | 0 |  |  |  |  |  |  |  |  |  |  |  |
| Fearful | 0.317 | 0.080 | 0.045 | 0.053 | 0.037 | 0 |  |  |  |  |  |  |  |  |  |  |
| Restless | 0.083 | 0.088 | 0.041 | 0.041 | 0.231 |  | 0 |  |  |  |  |  |  |  |  |  |
| Happy | 0.217 | 0.152 | 0.039 |  | 0.029 | 0.161 | 0.044 | 0 |  |  |  |  |  |  |  |  |
| Lonely |  |  |  | 0.296 |  |  |  | -0.040 | 0 |  |  |  |  |  |  |  |
| Stuck | 0.069 | 0.080 | 0.069 |  | 0.132 | 0.033 | 0.162 | 0.040 |  | 0 |  |  |  |  |  |  |
| IR |  |  |  | -0.023 |  |  |  | -0.025 |  |  | 0 |  |  |  |  |  |
| DR |  | -0.027 |  |  |  |  | -0.027 |  |  |  | 0.464 | 0 |  |  |  |  |
| TO |  |  | -0.029 |  |  |  |  |  |  | -0.032 |  | 0.044 | 0 |  |  |  |
| NA | -0.036 |  | -0.023 |  |  |  |  |  |  |  | 0.037 | 0.030 | 0.057 | 0 |  |  |
| PD |  |  |  |  |  |  |  |  |  |  |  | 0.019 |  | 0.037 | 0 |  |
| Sleep |  |  | -0.221 | -0.038 |  |  |  |  |  |  |  |  |  |  |  | 0 |

Lower triangle indicates within-person partial correlations between variables in the leftmost column and variables in the topmost row within the same measurement occasion, after controlling for other variables and all temporal relationships.

**Table S7.** Numeric results of Between-subject network for females.

|  | 1 | 2 | 3 | 4 | 5 | 6 | 7 | 8 | 9 | 10 | 11 | 12 | 13 | 14 | 15 | 16 |
| --- | --- | --- | --- | --- | --- | --- | --- | --- | --- | --- | --- | --- | --- | --- | --- | --- |
| Bothered | 0 |  |  |  |  |  |  |  |  |  |  |  |  |  |  |  |
| Distracted | -0.150 | 0 |  |  |  |  |  |  |  |  |  |  |  |  |  |  |
| Depressed | -0.161 |  | 0 |  |  |  |  |  |  |  |  |  |  |  |  |  |
| Drained | -0.208 |  |  | 0 |  |  |  |  |  |  |  |  |  |  |  |  |
| Hopeful | -0.003 |  |  | 0.118 | 0 |  |  |  |  |  |  |  |  |  |  |  |
| Fearful | -0.749 |  |  |  |  | 0 |  |  |  |  |  |  |  |  |  |  |
| Restless |  | 0.261 |  |  | 0.435 |  | 0 |  |  |  |  |  |  |  |  |  |
| Happy | -0.147 | 0.334 |  |  |  |  |  | 0 |  |  |  |  |  |  |  |  |
| Lonely |  |  |  | 0.601 |  | -0.100 | 0.141 |  | 0 |  |  |  |  |  |  |  |
| Stuck |  |  |  |  |  | 0.235 | 0.447 |  | -0.109 | 0 |  |  |  |  |  |  |
| IR |  | -0.101 |  |  |  |  |  |  | -0.071 |  | 0 |  |  |  |  |  |
| DR |  |  |  |  |  | 0.060 |  |  |  |  | 0.895 | 0 |  |  |  |  |
| TO |  | 0.102 |  |  |  |  | -0.097 |  |  |  |  |  | 0 |  |  |  |
| NA |  |  |  |  | 0.121 |  | -0.179 |  |  |  | 0.110 |  | 0.195 | 0 |  |  |
| PD |  | -0.100 |  |  |  |  |  |  |  |  | 0.143 |  | 0.456 | 0.185 | 0 |  |
| Sleep |  |  | -0.692 |  |  |  |  |  |  |  |  |  |  |  |  | 0 |

Lower triangle indicates between-person partial correlations between variables in the leftmost column and variables in the topmost row within the same measurement occasion, after controlling for other variables.

**Table S8.** Numeric results of Temporal network for males.

|  | 1 | 2 | 3 | 4 | 5 | 6 | 7 | 8 | 9 | 10 | 11 | 12 | 13 | 14 | 15 | 16 |
| --- | --- | --- | --- | --- | --- | --- | --- | --- | --- | --- | --- | --- | --- | --- | --- | --- |
| Bothered | 0.044 | 0.024 | 0.009 | 0.014 | 0.017 | 0.037 | 0.006 | 0.011 | 0.011 | -0.002 | -0.014 | 0.012 | 0.025 | -0.004 | 0.012 | 0.008 |
| Distracted | 0.009 | 0.036 |  | 0.008 | -0.009 | -0.001 | 0.016 | 0.006 | -0.003 | 0.003 | 0.005 | -0.001 | -0.021 | 0.024 | -0.001 | 0.005 |
| Depressed | 0.029 | 0.002 | 0.075 | -0.008 | 0.025 | 0.020 | 0.000 | 0.008 | 0.022 | -0.018 | -0.006 | 0.007 | 0.005 | -0.014 | 0.010 | -0.058 |
| Drained | 0.009 | 0.021 | 0.022 | 0.035 | 0.024 | 0.022 | 0.019 | 0.015 | 0.009 | 0.007 | -0.006 | 0.005 | -0.018 | -0.027 | -0.006 | -0.013 |
| Hopeful | 0.024 | 0.008 | 0.005 | 0.002 | 0.073 | -0.003 | 0.028 | 0.015 | 0.008 | 0.022 | 0.002 | -0.012 | -0.002 | 0.013 | 0.008 | 0.008 |
| Fearful | 0.029 | 0.005 | 0.007 | 0.002 | -0.017 | 0.033 | -0.005 | 0.002 | 0.009 | 0.007 | -0.004 | -0.015 | -0.008 | 0.005 | -0.007 | -0.010 |
| Restless | 0.009 | 0.016 | -0.007 | 0.021 | 0.017 | -0.003 | 0.046 | 0.009 | 0.014 | 0.024 | 0.006 | -0.007 | -0.014 | 0.001 | -0.003 | 0.001 |
| Happy | 0.001 | 0.002 | 0.001 | 0.007 | 0.015 | -0.009 | 0.003 | 0.003 | -0.001 | 0.022 | 0.019 | 0.009 | 0.009 | -0.017 |  | 0.004 |
| Lonely | 0.003 | 0.005 | 0.013 | 0.002 | -0.003 | 0.010 | 0.007 | 0.003 | 0.005 | -0.013 | 0.002 | -0.003 | 0.011 | 0.015 | 0.015 | -0.009 |
| Stuck | -0.009 | 0.006 | -0.002 | -0.001 |  | 0.015 | 0.015 | 0.011 | -0.004 | 0.031 | -0.012 | -0.009 | -0.018 | -0.012 | -0.004 | 0.007 |
| IR | -0.019 | 0.005 | -0.015 | 0.011 | -0.004 | -0.005 | -0.004 | -0.008 | 0.009 | -0.027 | 0.032 | 0.027 | 0.022 | 0.020 | 0.005 | 0.011 |
| DR | -0.001 | -0.021 | 0.000 | 0.001 | -0.001 | -0.013 | -0.025 | 0.005 | -0.019 | 0.011 | 0.030 | 0.049 | 0.018 | 0.029 | -0.011 | -0.011 |
| TO | 0.005 | -0.013 | 0.013 | -0.008 | -0.002 | 0.012 | -0.012 | 0.003 | -0.007 | -0.020 | -0.007 | 0.014 | 0.090 | 0.007 | -0.001 | 0.002 |
| NA | -0.001 | 0.013 | -0.003 | -0.003 | 0.005 | -0.002 | 0.018 | -0.007 | 0.009 | -0.016 | 0.031 | 0.041 | -0.009 | 0.053 | 0.018 | -0.006 |
| PD |  | 0.024 | 0.017 | 0.010 | 0.007 | 0.017 | 0.034 | 0.025 | -0.008 | 0.009 | 0.026 | 0.017 | 0.001 | 0.015 | 0.040 | 0.009 |
| Sleep | -0.006 | -0.014 | -0.027 | -0.015 | -0.001 | -0.005 | 0.001 | -0.001 | 0.009 | -0.014 | 0.017 | 0.005 | 0.018 | -0.028 | 0.003 | 0.084 |

The matrix shows how much each variable in the left column predicts the next lag-1 value of each variable in the top row. Diagonal numbers indicate edge weights of autoregressions.

**Table S9.** Numeric results of Contemporaneous network for males.

|  | 1 | 2 | 3 | 4 | 5 | 6 | 7 | 8 | 9 | 10 | 11 | 12 | 13 | 14 | 15 | 16 |
| --- | --- | --- | --- | --- | --- | --- | --- | --- | --- | --- | --- | --- | --- | --- | --- | --- |
| Bothered | 0 |  |  |  |  |  |  |  |  |  |  |  |  |  |  |  |
| Distracted | 0.201 | 0 |  |  |  |  |  |  |  |  |  |  |  |  |  |  |
| Depressed | 0.065 | 0.061 | 0 |  |  |  |  |  |  |  |  |  |  |  |  |  |
| Drained | 0.051 | 0.019 | 0.000 | 0 |  |  |  |  |  |  |  |  |  |  |  |  |
| Hopeful | 0.109 | 0.050 | 0.054 | 0.054 | 0 |  |  |  |  |  |  |  |  |  |  |  |
| Fearful | 0.284 | 0.085 | 0.049 | 0.052 | 0.022 | 0 |  |  |  |  |  |  |  |  |  |  |
| Restless | 0.067 | 0.085 | 0.041 | 0.030 | 0.227 | 0.016 | 0 |  |  |  |  |  |  |  |  |  |
| Happy | 0.195 | 0.147 | 0.039 | -0.005 | 0.036 | 0.149 | 0.043 | 0 |  |  |  |  |  |  |  |  |
| Lonely | 0.020 | -0.006 | -0.010 | 0.293 | -0.001 | -0.018 | 0.024 | -0.041 | 0 |  |  |  |  |  |  |  |
| Stuck | 0.047 | 0.077 | 0.062 | 0.015 | 0.128 | 0.040 | 0.170 | 0.038 | -0.008 | 0 |  |  |  |  |  |  |
| IR | -0.017 | -0.005 | -0.002 | -0.019 | 0.021 | -0.007 | -0.001 | -0.015 | -0.016 | 0.004 | 0 |  |  |  |  |  |
| DR | 0.012 | -0.020 | -0.013 | 0.005 | -0.020 | -0.014 | -0.024 | -0.001 | 0.000 | 0.002 | 0.464 | 0 |  |  |  |  |
| TO | -0.001 | -0.010 | -0.016 | 0.000 | -0.015 | 0.026 | -0.009 | -0.011 | -0.011 | -0.028 | 0.013 | 0.040 | 0 |  |  |  |
| NA | -0.006 | -0.004 | -0.021 | -0.015 | -0.006 | -0.016 | -0.001 | -0.012 | -0.001 | -0.017 | 0.039 | 0.029 | 0.054 | 0 |  |  |
| PD | -0.011 | 0.006 | 0.015 | -0.005 | 0.005 | 0.001 | -0.001 | -0.005 | -0.011 | -0.003 | -0.008 | 0.022 | 0.017 | 0.042 | 0 |  |
| Sleep | -0.006 | 0.001 | -0.215 | -0.033 | -0.013 | -0.008 | 0.000 | 0.024 | -0.007 | -0.003 | 0.009 | -0.012 | -0.001 | 0.011 | -0.002 | 0 |

Lower triangle indicates within-person partial correlations between variables in the leftmost column and variables in the topmost row within the same measurement occasion, after controlling for other variables and all temporal relationships.

**Table S10.** Numeric results of Between-subject network for males.

|  | 1 | 2 | 3 | 4 | 5 | 6 | 7 | 8 | 9 | 10 | 11 | 12 | 13 | 14 | 15 | 16 |
| --- | --- | --- | --- | --- | --- | --- | --- | --- | --- | --- | --- | --- | --- | --- | --- | --- |
| Bothered | 0 |  |  |  |  |  |  |  |  |  |  |  |  |  |  |  |
| Distracted | 0.320 | 0 |  |  |  |  |  |  |  |  |  |  |  |  |  |  |
| Depressed | -0.017 | 0.101 | 0 |  |  |  |  |  |  |  |  |  |  |  |  |  |
| Drained | 0.279 | -0.087 | 0.031 | 0 |  |  |  |  |  |  |  |  |  |  |  |  |
| Hopeful | 0.270 | -0.124 | 0.076 | -0.011 | 0 |  |  |  |  |  |  |  |  |  |  |  |
| Fearful | 0.456 | -0.079 | 0.068 | 0.131 | 0.027 | 0 |  |  |  |  |  |  |  |  |  |  |
| Restless | -0.030 | 0.261 | -0.084 | 0.096 | 0.454 | 0.088 | 0 |  |  |  |  |  |  |  |  |  |
| Happy | 0.374 | 0.304 | 0.066 | -0.115 | -0.139 | 0.192 | -0.013 | 0 |  |  |  |  |  |  |  |  |
| Lonely | -0.125 | 0.149 | -0.056 | 0.624 | 0.103 | -0.115 | -0.029 | 0.007 | 0 |  |  |  |  |  |  |  |
| Stuck | 0.128 | 0.080 | 0.111 | -0.066 | -0.008 | 0.109 | 0.324 | -0.036 | -0.024 | 0 |  |  |  |  |  |  |
| IR | 0.134 | -0.069 | -0.073 | -0.050 | -0.058 | -0.027 | -0.055 | -0.008 | 0.010 | -0.047 | 0 |  |  |  |  |  |
| DR | -0.114 | 0.002 | 0.112 | 0.053 | 0.018 | 0.118 | 0.035 | -0.065 | -0.067 | 0.096 | 0.887 | 0 |  |  |  |  |
| TO | -0.140 | 0.259 | -0.094 | 0.075 | 0.021 | 0.048 | -0.050 | -0.100 | -0.008 | 0.006 | -0.003 | 0.010 | 0 |  |  |  |
| NA | -0.070 | -0.070 | 0.047 | 0.034 | 0.166 | -0.032 | -0.185 | 0.143 | -0.070 | 0.022 | 0.101 | 0.016 | 0.260 | 0 |  |  |
| PD | 0.112 | -0.164 | -0.037 | -0.011 | 0.002 | -0.054 | -0.019 | 0.068 | -0.030 | -0.059 | 0.088 | 0.069 | 0.463 | 0.106 | 0 |  |
| Sleep | -0.012 | 0.061 | -0.719 | -0.027 | 0.091 | -0.016 | -0.065 | 0.028 | -0.046 | 0.070 | -0.064 | 0.092 | -0.066 | -0.004 | -0.019 | 0 |

Lower triangle indicates between-person partial correlations between variables in the leftmost column and variables in the topmost row within the same measurement occasion, after controlling for other variables.
