# Supplementary figures and images for: Dynamic Interactions Among Sleep Duration, Cognitive Function, and Depressive Symptoms in Middle-Aged and Older Chinese Adults: Temporal Network Analysis From CHARLS
Source: JMIR Aging. 2025 Sep 16;8:e76210. doi: 10.2196/76210 (PMC12440260; doi:10.2196/76210)

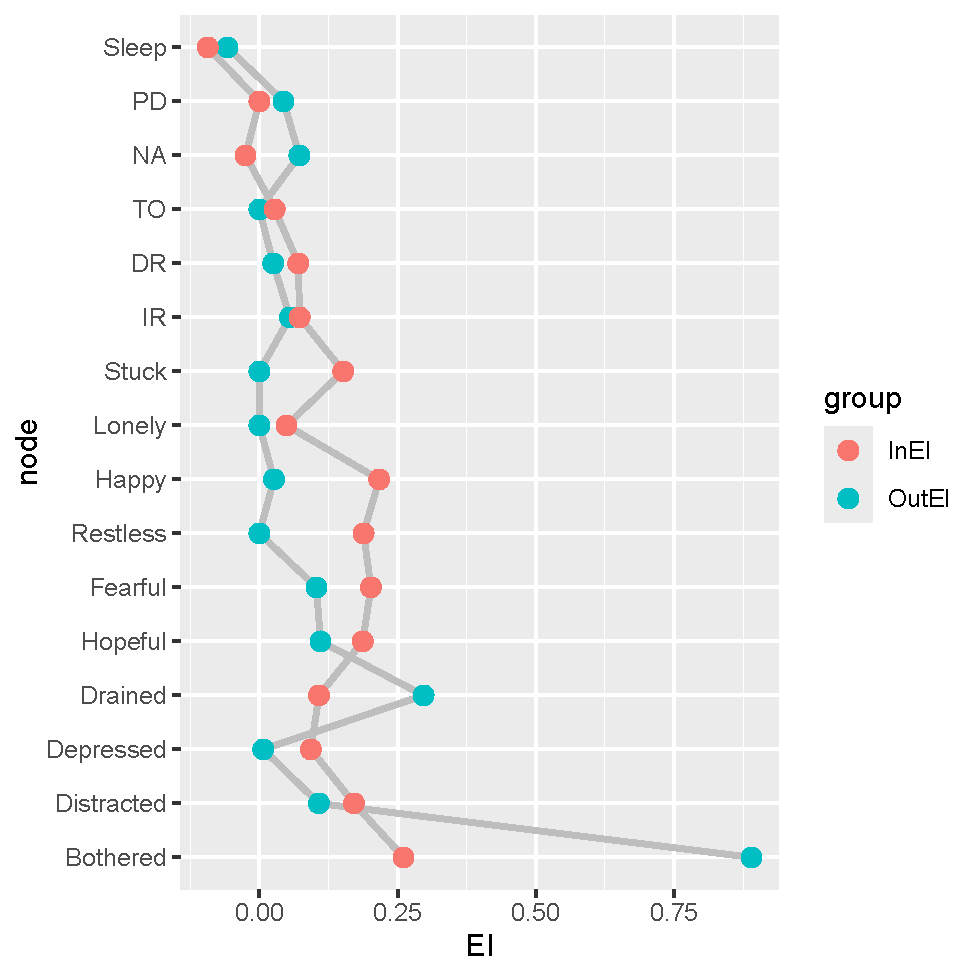

Supplement: Multimedia Appendix 2 [file aging-v8-e76210-s002.png]

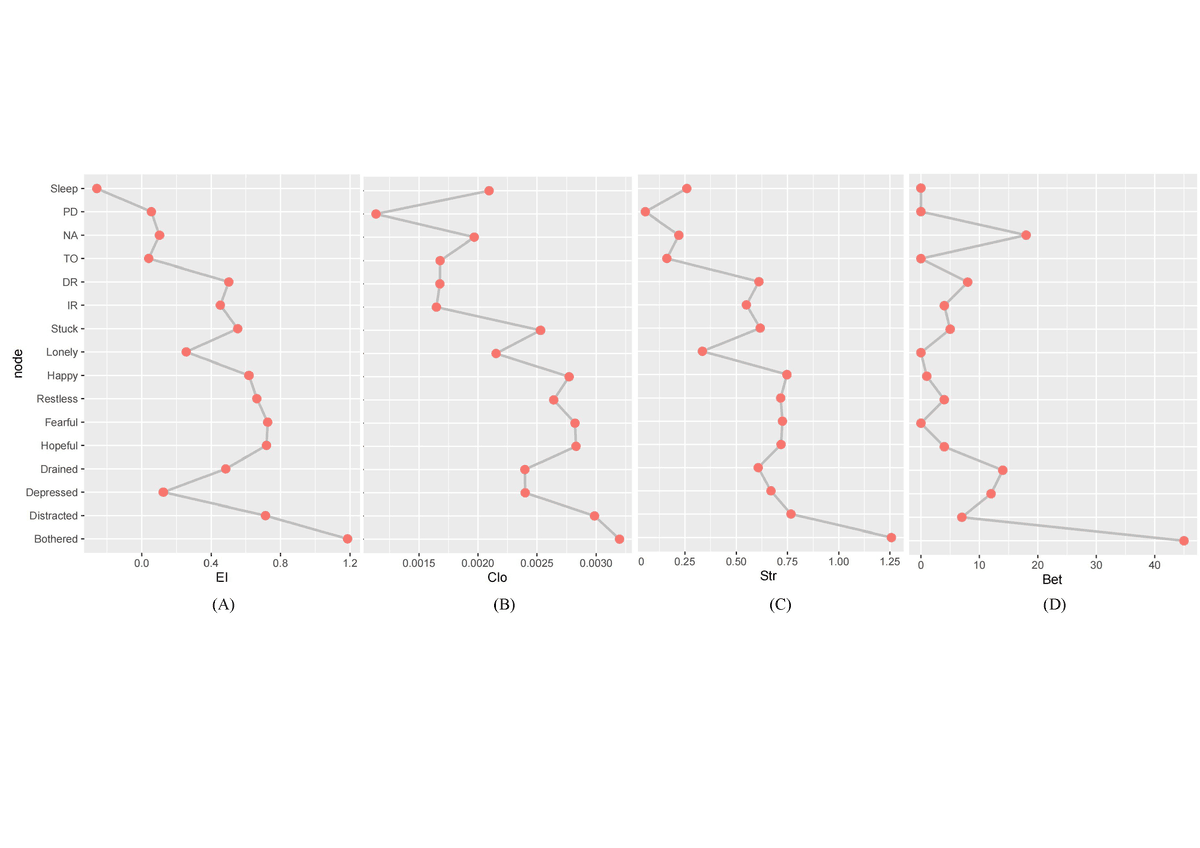

Supplement: Multimedia Appendix 3 [file aging-v8-e76210-s003.png]

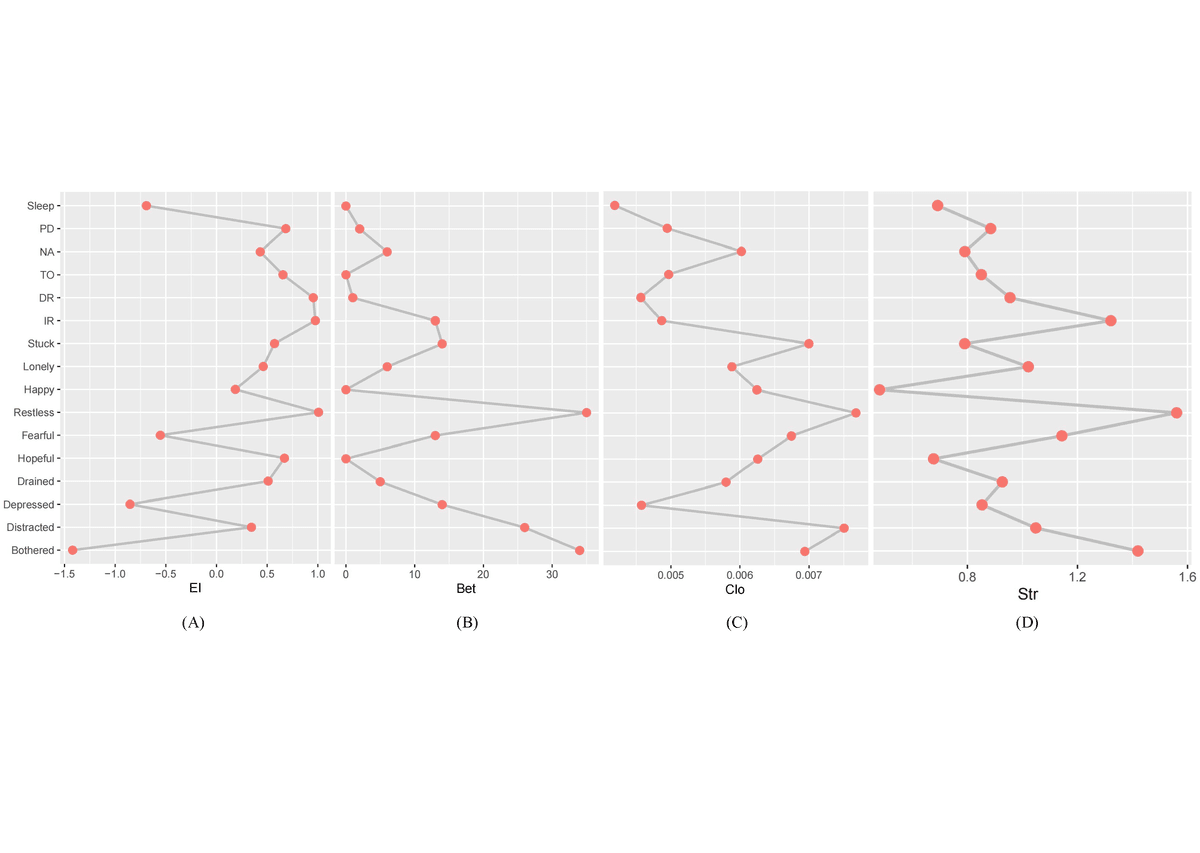

Supplement: Multimedia Appendix 4 [file aging-v8-e76210-s004.png]

Temporal Network

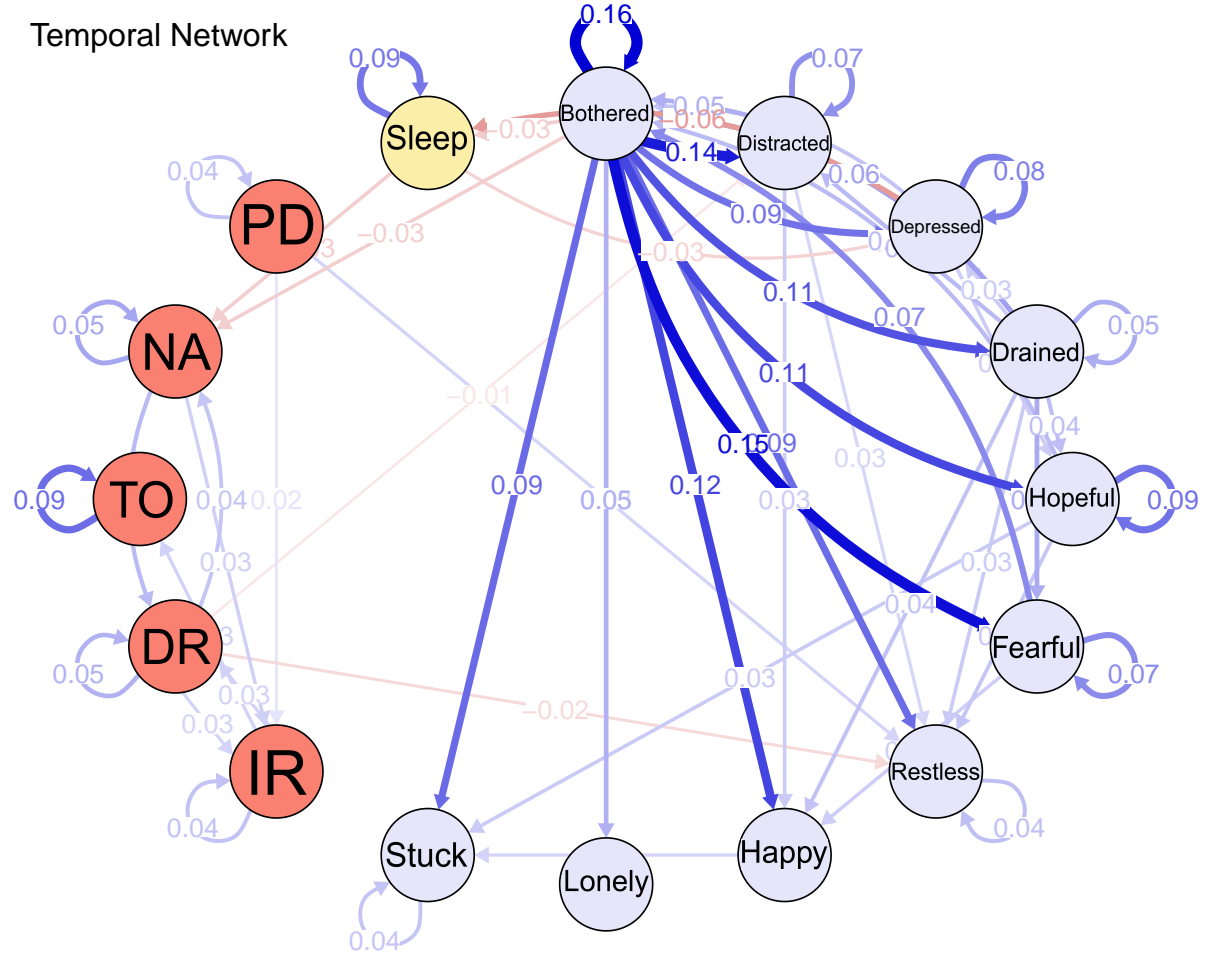

Supplement: Multimedia Appendix 5 [file aging-v8-e76210-s005.pdf]

Temporal Network

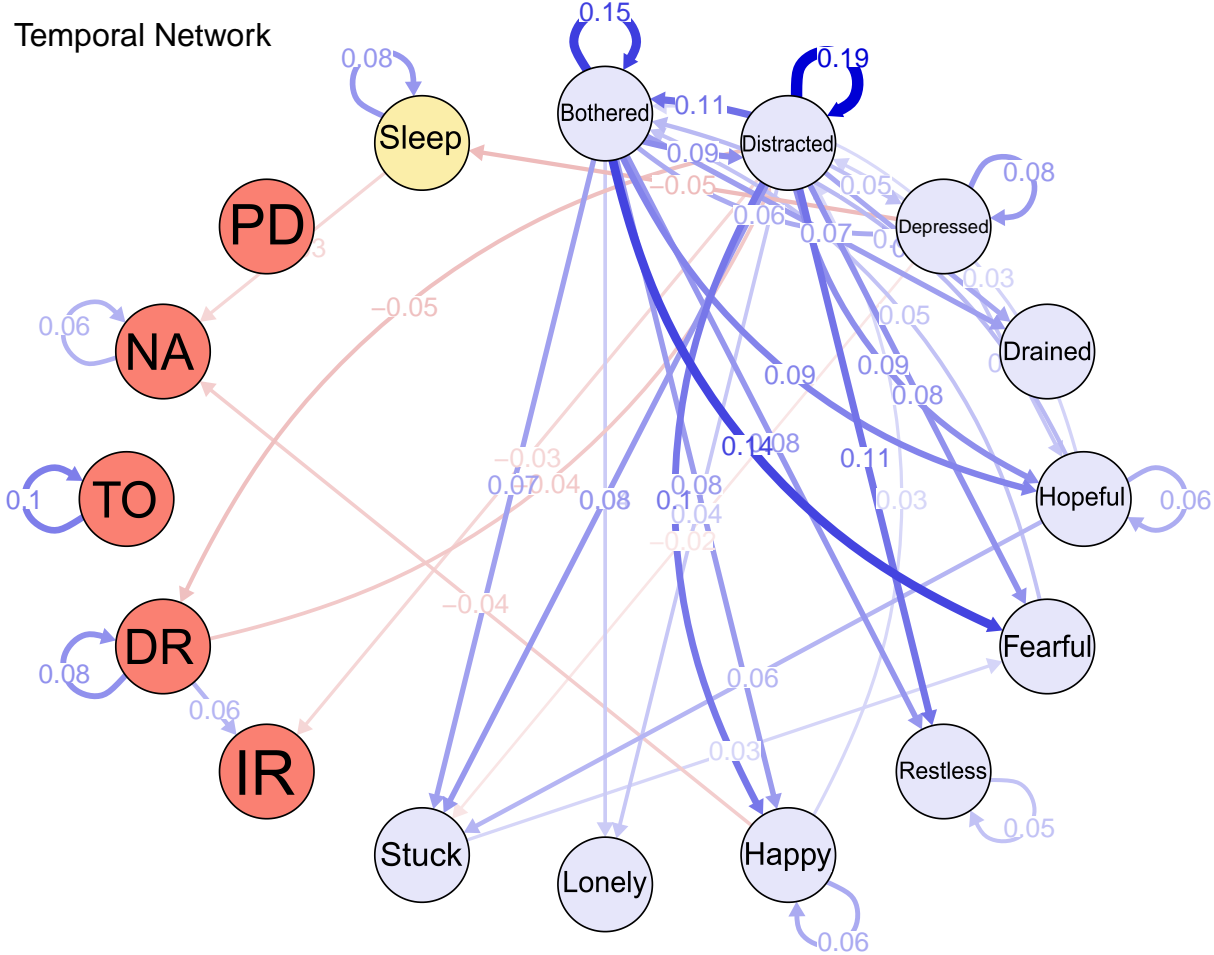

Supplement: Multimedia Appendix 6 [file aging-v8-e76210-s006.pdf]

## Contemporaneous Network

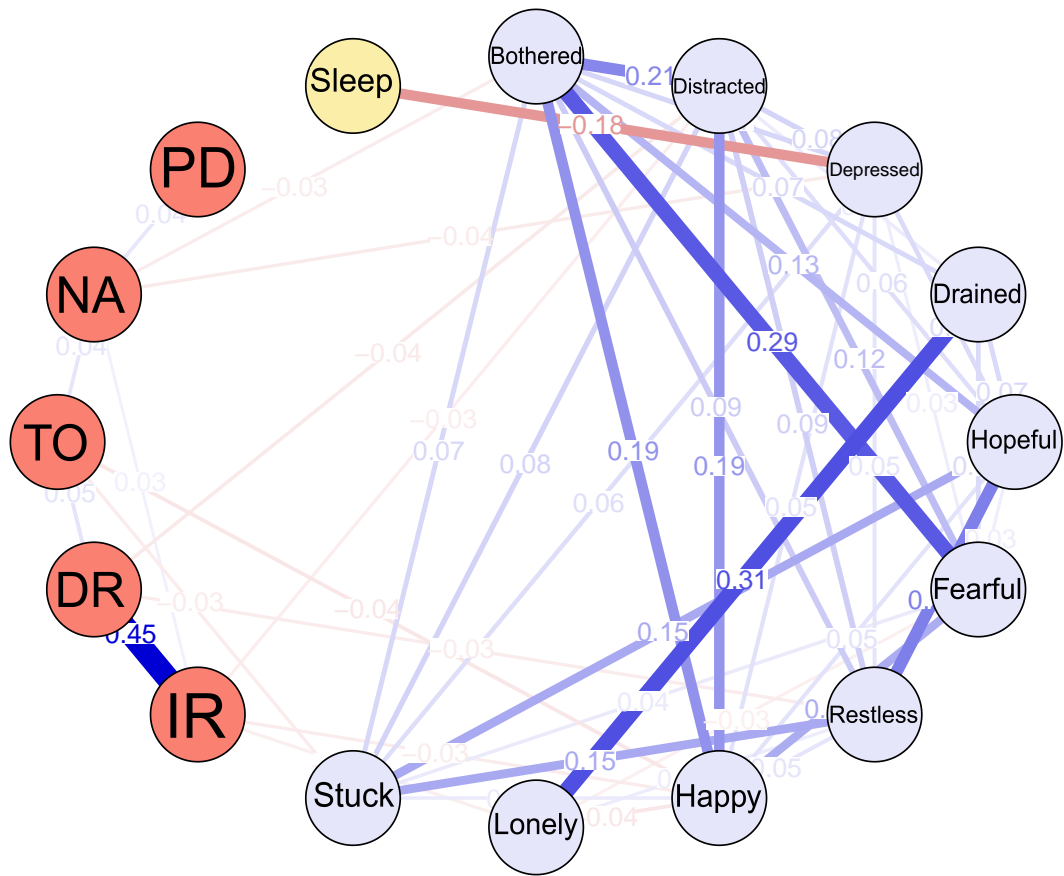

Supplement: Multimedia Appendix 7 [file aging-v8-e76210-s007.pdf]

Contemporaneous Network

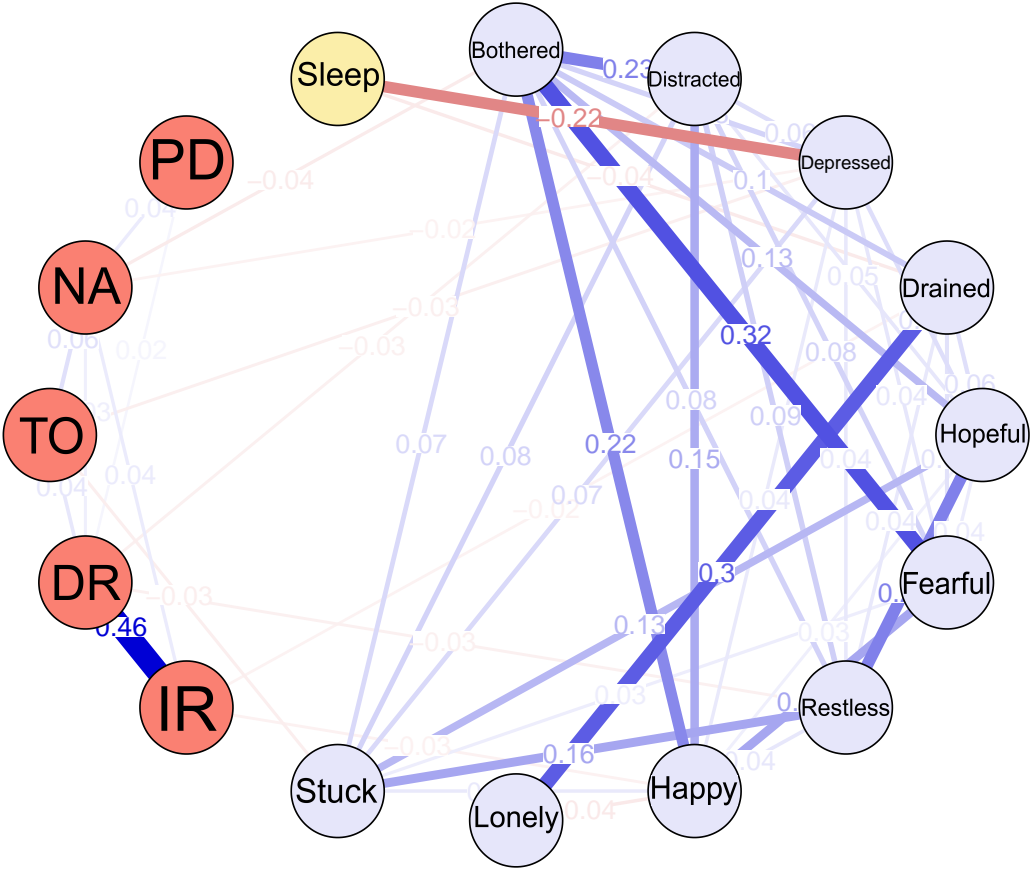

Supplement: Multimedia Appendix 8 [file aging-v8-e76210-s008.pdf]

Between-subject Network

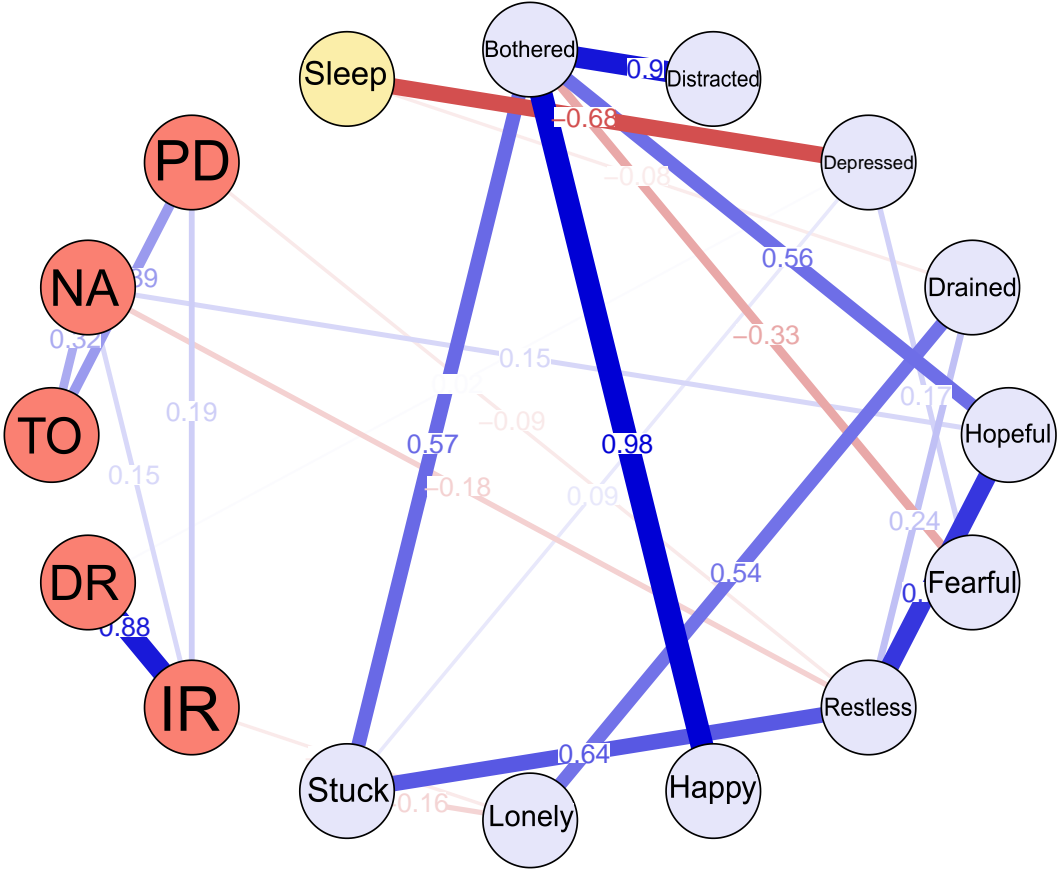

Supplement: Multimedia Appendix 9 [file aging-v8-e76210-s009.pdf]

Between-subject Network

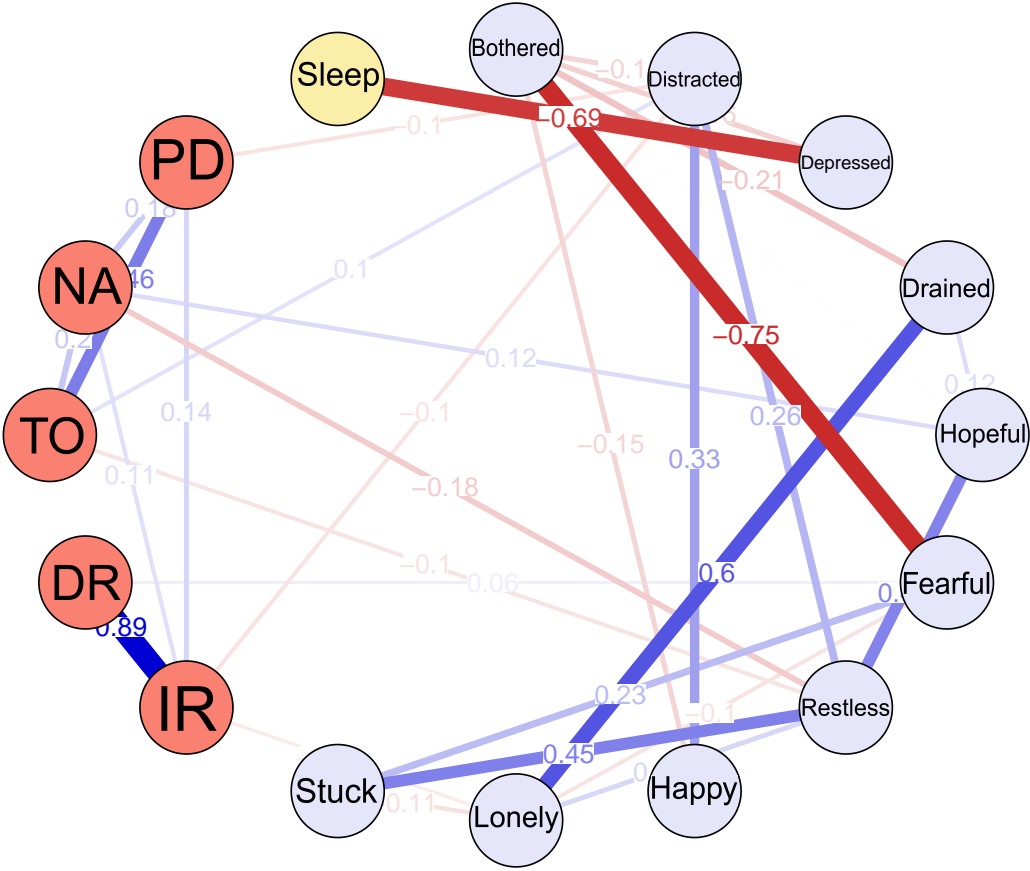

Supplement: Multimedia Appendix 10 [file aging-v8-e76210-s010.pdf]
